# The origin of animals: an ancestral reconstruction of the unicellular-to-multicellular transition

**DOI:** 10.1098/rsob.200359

**Published:** 2021-02-24

**Authors:** Núria Ros-Rocher, Alberto Pérez-Posada, Michelle M. Leger, Iñaki Ruiz-Trillo

**Affiliations:** ^1^ Institut de Biologia Evolutiva (CSIC-Universitat Pompeu Fabra), Passeig Marítim de la Barceloneta 37-49, 08003 Barcelona, Catalonia, Spain; ^2^ Centro Andaluz de Biología del Desarrollo (CSIC-Universidad Pablo de Olavide), Carretera de Utrera Km 1, 41013 Sevilla, Andalusia, Spain; ^3^ Departament de Genètica, Microbiologia i Estadística, Institut de Recerca de la Biodiversitat, Universitat de Barcelona, Avinguda Diagonal 643, 08028 Barcelona, Catalonia, Spain; ^4^ ICREA, Passeig Lluís Companys 23, 08010 Barcelona, Catalonia, Spain

**Keywords:** multicellularity, animal origins, evolutionary transitions, cell-type evolution, Holozoa

## Abstract

How animals evolved from a single-celled ancestor, transitioning from a unicellular lifestyle to a coordinated multicellular entity, remains a fascinating question. Key events in this transition involved the emergence of processes related to cell adhesion, cell–cell communication and gene regulation. To understand how these capacities evolved, we need to reconstruct the features of both the last common multicellular ancestor of animals and the last unicellular ancestor of animals. In this review, we summarize recent advances in the characterization of these ancestors, inferred by comparative genomic analyses between the earliest branching animals and those radiating later, and between animals and their closest unicellular relatives. We also provide an updated hypothesis regarding the transition to animal multicellularity, which was likely gradual and involved the use of gene regulatory mechanisms in the emergence of early developmental and morphogenetic plans. Finally, we discuss some new avenues of research that will complement these studies in the coming years.

## Graphical abstract

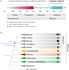

## 1. An overview of animal origins

Animals (Metazoa) are among the major groups of complex multicellular organisms. They rely on a wide variety of differentiated cell types that are spatially organized within physiological systems. At the same time, animal cells perform specialized functions, and thus evolved the capacity to integrate and coordinate them using tightly regulated developmental programmes. However, we still do not know which genetic and mechanistic factors underpinned the origin and evolution of animal multicellularity.

**Figure 1.**
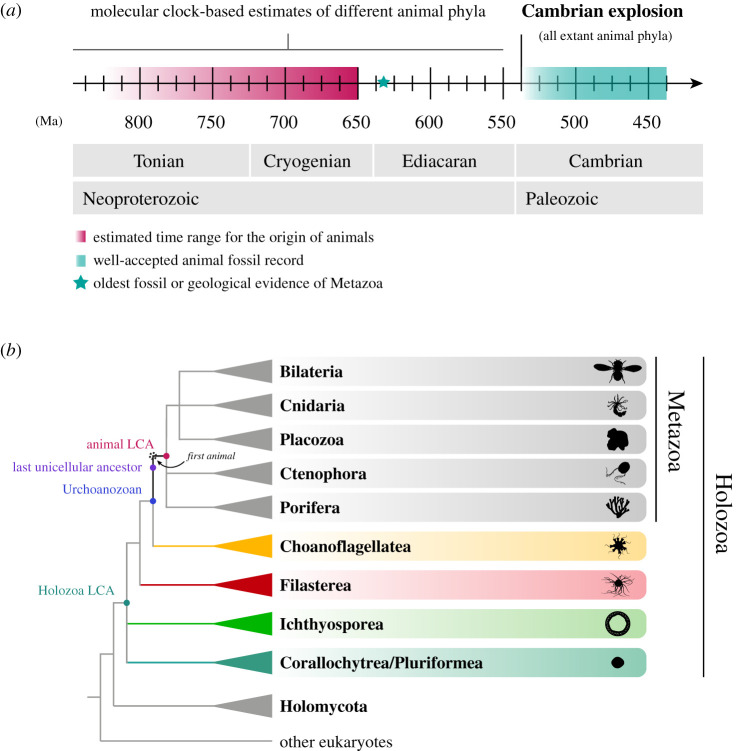
Phylogenetic classification of animals and their unicellular relatives. (*a*) A timeline of different events during early animal evolution. The transition to animal multicellularity, and therefore the origin of the first animals, occurred sometime at the end of the Tonian period, according to molecular clock estimates. The oldest fossil or geological evidence of recognizable animals dates back to the Ediacaran period, with molecular clocks extending the emergence of different animal phyla back to the Cryogenian [15–17]. Time units are million years ago (Ma). (*b*) Cladogram representing the major clades of the tree of animals and the major groups of unicellular relatives of animals: choanoflagellates, filastereans, ichthyosporeans and corallochytreans/pluriformeans. Coloured nodes indicate different ancestors that we can reconstruct and that are important to understand the transition to animal multicellularity; the highlighted internal branch (from the Urchoanozoan to the animal LCA) indicates the animal stem (see box 1; LCA = last common ancestor). Uncertain positions within the animal tree [18–23] and within Holozoa [24–26] are represented with polytomies.

All extant animals living today diversified from a common multicellular ancestor, also known as the last common ancestor (LCA) of animals or the animal LCA (box 1). The animal LCA evolved from a single-celled ancestor more than 600 million years ago (Ma), transitioning from a unicellular ancestral state to complex multicellularity (box 1, figure 1*a*). By comparing the nature of these two ancestral states—the last unicellular ancestor and the animal LCA—we can uncover the major changes that drove the transition to animal multicellularity and create new, testable hypotheses about the origin of animals. The questions are, then: What were these two animal ancestors like? Was the last unicellular ancestor very simple, or was it quite complex, establishing the foundations for cell differentiation and multicellularity? And what was the animal LCA like? Was it simple, gradually acquiring new developmental capabilities while diversifying into different body plans, or was it already complex, creating the genetic conditions for a successful animal diversification?

Box 1. **Terminology used in this review.**

*Last common ancestor of animals (animal LCA)*: The ancestral stage from which all animal phyla living today radiated. Reconstructed from features present in, and shared by, extant animals. Undoubtedly presenting all the features shared by all animals, including complex, coordinated multicellularity. Therefore, it can be classified as an animal.

*Last unicellular ancestor of animals*: The single-celled ancestor immediately preceding the emergence of the first animal.

*Complex multicellularity:* An assembly of cells displaying a three-dimensional organization and complex body plans arising from a centralized developmental programme.

*Simple multicellularity*: An assembly of cells, including filaments, clusters, balls, sheets or mats, that arise via mitotic cell division from a single progenitor or by aggregation of independent cells. Simple multicellularity can be found in prokaryotes and eukaryotes.

*First animal*: First multicellular ancestor of all extant animals. Partly reconstructed from features shared between early diverging animal lineages (i.e. sponges, ctenophores, placozoans and cnidarians), even if these features are absent from bilaterians. This ancestor lived subsequent to changes that led to the foundations of complex multicellularity in animals and is unlikely to be the same as the animal LCA.

*Animal stem*: The evolutionary lineage leading to all animals, from the common ancestor of animals and choanoflagellates (Urchoanozoan) to the animal LCA. The subsequent transition from unicellularity to multicellularity occurred along the animal stem lineage.

*Urmetazoa*: A term used in the literature, that is variously defined as the first animal, the animal LCA, or as an amalgam of the two. To avoid confusion, we do not use this term in this review

*Urchoanozoan*: The last common ancestor of animals and choanoflagellates. It may or may not be the same as the last unicellular ancestor of animals.

*Holozoa*: Eukaryotic group comprising animals, choanoflagellates, filastereans, ichthyosporeans and corallochytreans/pluriformeans. The largest clade including *Homo sapiens* but not *Neurospora crassa* [1].

*Last common ancestor of Holozoa (Holozoa LCA)*: The ancestor shared by Metazoa, Choanoflagellatea, Filasterea, Ichthyosporea and Corallochytrea/Pluriformea.

*Metacell*: In single-cell genomics, a subgroup of homogeneous scRNASeq profiles with only local variance relative to the total dataset, useful for clustering and quantitative gene expression analyses [2]. Ultimately, it can be related to certain cell types, but only upon experimental validation.

*Cell type*: In its simplest definition, a cell type was defined as a unit of classification to distinguish forms of cells according to different morphologies or phenotypes. Cell types are often related to different germ layers during the formation of the embryo, with nerve and epithelial cells coming from the ectoderm, muscle and blood cells from the mesoderm, and gut cells from the endoderm [3–5]. Whereas vertebrate cell types are often defined by their committed fate and being unable to de-differentiate, cells from early branching animals are known to transdifferentiate and change their cell types [6]. This has led to numerous revisions of the concept at the functional, developmental, and even molecular (gene expression) level. Here, we use the term ‘cell type’ as ‘a classification unit based on the combined observations of a cell morphology and gene expression profile, which is driven by a gene regulatory network and can be repeatedly found within the context of a species'. These cell types can be part of either a spatially or a temporally integrated life cycle.

*Aggregative multicellularity*: One of the two known mechanisms for evolving multicellularity. Aggregative multicellularity is the result of two or more independent and genetically distinct cells binding to or aggregating with each other. The resulting multicellular structure consists of a heterogeneous population of cells, and it is often formed for the purpose of reproduction and dispersion [7–9]. It has evolved repeatedly across different eukaryotic lineages [10–14].

*Clonal multicellularity*: One of the two known mechanisms for evolving multicellularity. Clonal multicellularity arises through successive rounds of cell division from a single founder cell (spore or zygote) with incomplete cytokinesis (i.e. division of the cytoplasm of the parental cell into two daughter cells). It has appeared on fewer occasions and is responsible for the best-known radiations of complex multicellular life forms in the tree of life: land plants, fungi and animals.

Recent data from a broad representation of animal species, especially from non-bilaterian animals (sponges, ctenophores, placozoans and cnidarians), and also from unicellular species related to animals, have enabled us to better answer these questions. Their genome content, gene regulatory capabilities and biological features can be compared to reconstruct the cellular foundations of animal evolution and infer the minimal genomic complexity of both the last unicellular ancestor of animals and the animal LCA. Moreover, the advent of sequencing technologies, such as single-cell omics, and the development of genetic tools among unicellular relatives of animals are opening new avenues of research for gene function studies, pointing to an ever-expanding breadth of exciting questions that will complement these inferences from a functional and biological perspective.

In this review, we provide an updated reconstruction of these two evolutionary stages that are key to better understanding the transition to animal multicellularity: 1) the last unicellular ancestor of animals and 2) the animal LCA. We summarize current knowledge on the genetic toolkit, cell-type diversity and ecological context of these ancestors, inferred by comparative genomic analyses between animals with their closest unicellular relatives and between the earliest branching animals and those radiating later. On this basis, we propose an updated hypothesis to explain the transition to animal multicellularity, stressing that animal foundations were laid before the origin of animals and that the gradual complexification of genetic regulatory mechanisms was key to the progressive acquisition of animal axial cell patterning and cell-type identity. Finally, we discuss some of the research areas that we predict will be key to studying animal origins in the coming years.

### 1.1. Phylogenetic framework of animals and their unicellular relatives

The reconstruction of any evolutionary event relies on a well-supported phylogenetic framework. Thus, to infer the genomic and biological features of the last unicellular ancestor of animals and the animal LCA, the first step is to define the evolutionary relationships between animals and between animals and their closest relatives. The animal tree of life has been deeply studied [18,27–31] (see [32] for a review), yet a consistent, well-supported phylogeny remains elusive. Some areas of uncertainty remain, especially around the root of Metazoa, due largely to choices made in different phylogenomic analyses, such as the genes selected, taxon sampling used, the assembly of the phylogenomic data matrix or the model of sequence evolution [18,31–33]. The latter can contribute to violations of model assumptions, known as systematic errors (e.g. long-branch attraction artefacts); these may also impact animal tree reconstruction [31]. This lack of consensus on relationships between the earliest branching Metazoa [18,19,31,33,34] has hindered the reconstruction of certain metazoan traits [33,35]. For instance, uncertainty regarding the position of Ctenophora or Porifera as the sister group of all other animals has led to continued debate regarding the origin and evolution of the nervous system [18–23,33,36–40]. Nonetheless, the robustness of other positions in the animal phylogeny allow us to infer many other features of the animal LCA [33].

Until recently, we knew very little about the tree of life surrounding animals, especially because a well-supported phylogeny relies on the availability of well-annotated genome-scale data and the placement of key taxa. In the last decade, the genome sequencing of several unicellular species has improved the phylogenetic framework of animals and their unicellular relatives [24,25,41–45]. Now we know that animals are closely related to a heterogeneous assembly of unicellular lineages known as unicellular holozoans, which together comprise the Holozoa clade within the eukaryotic group Opisthokonta (figures 1*b* and 3; box 1) [25,46–51]. The closest unicellular lineage to animals is Choanoflagellatea, a group of more than 250 species of spherical/ovoid heterotrophic flagellates (figure 1*b*) [52]. Their representatives, the choanoflagellates, have been linked to animals for over a century because of their morphological resemblance to *choanocytes*, a specific cell type of sponges [53]. This similarity, together with the confirmation from molecular phylogenies of their position as a sister group of animals (figures 1*b* and 3*a*,*b*) [47,48,52,54–59], has historically given rise to hypotheses of animals evolving from a choanoflagellate-like ancestor [60–63]. Molecular phylogenies have confirmed two additional independent lineages within Holozoa: Filasterea and Ichthyosporea (figure 1*b*). Filasterea is the sister group of Choanoflagellatea and Metazoa, and is so far known to include only five amoeboid and amoeboflagellate species (figures 1*b* and 3*c*,*d*) [25,26,48–50,55,64–71]. Ichthyosporea is the sister group to the rest of Holozoa and is a diverse group of around 40 osmotrophic and saprotrophic protists (figure 1*b* and 3*e*,*f*) [72–82]. Nevertheless, the addition of new species has left some uncertainties in the holozoan phylogeny, which appears to be highly sensitive to taxonomic sampling.

One open question concerns the position of the free-living osmotroph *Corallochytrium limacisporum* (figures 1*b* and 3*g*) [83]. *Corallochytrium* was previously classified as the sister group to Ichthyosporea, forming a monophyletic group named Teretosporea [24,25]. However, recent analyses including the newly described predatory flagellate *Syssomonas multiformis* (figure 3*h*) [26,70] grouped *Corallochytrium* and *Syssomonas* together in a new independent clade named Pluriformea, which branches between Filasterea and Ichthyosporea (figure 1*b*) [26]. A similar case concerns the unresolved position of the recently discovered *Tunicaraptor unikontum,* another predatory flagellate closely related to animals [84]. Depending on the taxon sampling used, *T. unikontum* may be sister to filastereans, Filozoa (which includes the filasterean–choanoflagellate–animal group), or it may be the earliest branching holozoan lineage [84]. Environmental surveys have also identified other putative new species falling within or related to different unicellular holozoan clades and even a potential novel lineage [85–93]. This indicates that there is still a substantial hidden diversity within the Holozoa clade, which may affect our reconstruction of the evolution of certain traits along the Holozoa stem. We expect future studies will improve our understanding of unicellular holozoan diversity and clarify the evolutionary relationships of the tree surrounding animals. Nevertheless, despite the previously mentioned conundrums in the Holozoa phylogeny, we can still make inferences based on the current data that we review in the following sections.

## 2. Reconstruction of the last unicellular ancestor of animals and the last common ancestor of animals

Under the Holozoa phylogenetic framework we can compare the genomic and biological features between unicellular holozoans and animals and reconstruct the two key evolutionary stages from which animals originated: the last unicellular ancestor of animals and the animal LCA (see box 2 for clarification).

Box 2. **Was the first animal similar to the animal LCA?**

The shared common multicellular ancestor from which all extant animals diversified (the animal LCA) may have not been the same as the first animal (box 1). The first animal was the first multicellular ancestor of all extant animals, and likely gave rise to other lineages that subsequently became extinct prior to the divergence of all modern animal lineages from the animal LCA. Despite research being so far limited to the reconstruction of the animal LCA (and the different unicellular ancestors of animals), we can partly reconstruct the first animal based on our current knowledge of the animal LCA and also from features shared between early diverging animals. For instance, we can infer that the genetic toolkit of the first animal was very rich in genes related to metazoan innovations, ranging from the cellular foundations of epithelial-like layers to neuron-like signalling cells and occurrence of muscle-like contractile cells. Many animal-specific pathways and mechanisms were thus largely complete in the animal LCA (similar to the observations about the cnidarian–bilaterian LCA by Putnam *et al*. [94]), suggesting that they were also present in previous ancestral states, possibly even in the first animals (figure 1, box 1). Similarly, based on our inferences of cell-type diversity in the animal LCA, those ancestors prior to the animal LCA likely had the ability to regulate cell differentiation by means of hierarchical TF networks and distal regulation in different cells within the multicellular collective, which translates to a certain degree of spatial cell differentiation possibly present in the first animals. Rather than a drastic bloom of innovations, it is likely that gene expansion, co-option, increased regulatory sophistication and a transition from temporal to spatial gene regulation had a crucial impact on the gradually increasing complexity of the first animals ([95], and references within).

Currently, phylogenomic studies and analysis for the reconstruction of animal ancestors are limited by the data available for such comparisons. For instance, genomic data on early branching animals is limited to a handful of species, which may or may not be good representatives due to gene loss and rapid evolution. Likewise, our findings would be biased towards the assumption of numerous innovations in the animal lineage unless we include other lineages in our comparisons. For these reasons, studying the origin and evolution of animals requires us to sequence more early branching animal genomes, and just as importantly, to expand our focus to other lineages outside Metazoa.

### 2.1. Reconstruction of the genomic features of the last unicellular ancestor of animals and the last common ancestor of animals

#### 2.1.1. The genetic toolkit of the last unicellular ancestor of animals

The nature of the last unicellular ancestor of animals can only be reconstructed through comparative studies between animals and their closest extant unicellular relatives, the unicellular holozoans. In the last decade, multiple omics-scale datasets have been generated from a broad representation of unicellular holozoan species. We currently have 11 complete genomes at our disposal [24,25,41–45] and around 30 transcriptomes and proteomes of several species, including representatives of each unicellular holozoan lineage [24–26,42,45,51,84,96–101]. These datasets have allowed us to identify the genomic features that are shared between extant unicellular holozoans and animals, which are thus inferred to be present in their last unicellular common ancestor.

**Figure 2.**
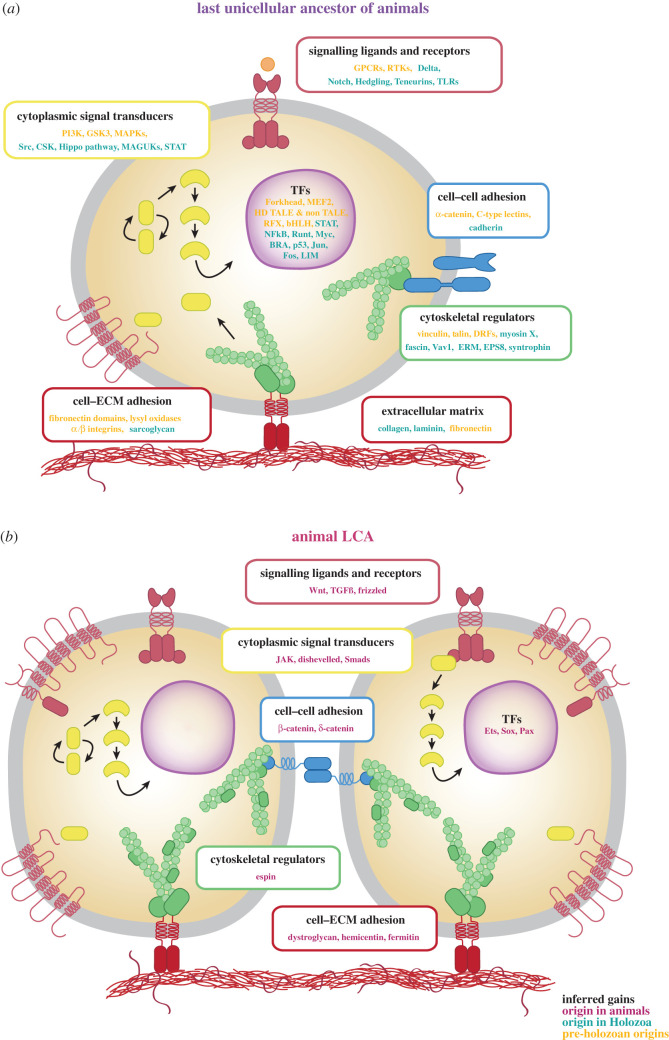
An inferred gene repertoire of the last unicellular ancestor and the last common ancestor of animals. (*a*) The reconstruction of the last unicellular ancestor of animals is based on the presence of key metazoan genes in the genomes of unicellular relatives of animals. (*b*) Inferred gains present in the last common ancestor (LCA) of animals. Yellow indicates genes that originated prior to the emergence of the Holozoa LCA (pre-holozoan origins); green, genes that originated in Holozoa prior to the animal LCA (Holozoa origins); red, animal-specific genes that originated at the root of animals (animal origins). bHLH, basic helix–loop–helix transcription factors; BRA, Brachyury; CSK, C-terminal Src kinase; DRFs, diaphanous-related formins; EPS8, epidermal growth factor receptor kinase substrate 8; ERM, Ezrin–Radixin–Moesin proteins; GPCRs, G protein-coupled receptors; GSK3, glycogen synthase kinase 3; HD, homeodomain; MAGUKs, membrane-associated guanylate kinases; MAPKs, mitogen-activated protein kinases; MEF2, myocyte-specific enhancer factor 2; NF-κB, nuclear factor-κB; PI3 K, phosphatidylinositol 3-kinase; RFX, regulatory factor X transcription factors; RTKs, receptor tyrosine kinases; STAT, signal transducer and activator of transcription; TALEs, three amino acid loop extensions; TFs, transcription factors; TGFß, transforming growth factor beta. Data from [24,26,44,45,97,102,105–121].

Strikingly, the genomes of extant unicellular holozoans indeed encode a large repertoire of genes that are homologous to genes critical for multicellularity-related functions in animals [24–26,41,42,44,45,97,98,100–104]. These include genes related to cell adhesion, signalling pathways and transcriptional regulation (figure 2*a*) [95,122,123]. For instance, a rich repertoire of genes related to cell adhesion in animals is found in the genomes of several unicellular holozoans. These include key genes mediating animal cell–cell adhesion, such as cadherin domain-containing proteins or C-type lectins, which are present in choanoflagellates and have a patchy distribution in other holozoans [84,97,105,124,125]. Integrins and associated scaffolding proteins, which mediate animal cell–extracellular matrix adhesion, are present in filastereans, ichthyosporeans, *C. limacisporum, S. multiformis* and *T. unikontum* [26,84,97,98,103,126]. Some choanoflagellate species also possess a small subset of the integrin adhesome system [97,98,103]. Moreover, other structural remodelling proteins, such as fascin or Ezrin–Radixin–Moesin and some basal lamina elements (i.e. collagen, laminin and fibronectin), are present in a few unicellular holozoan species [84,98,106]. Choanoflagellates and *T. unikontum* also encode several domains with affinity to the animal Ig-like domain families [41,84,97]. Altogether, this indicates that several genes from the animal cell adhesion machinery were already present in the last unicellular ancestor of animals (figure 2*a*).

The genomes of unicellular holozoans also encode homologues of key metazoan intracellular signalling components related to cell–cell communication, immunity and environmental signal/response pathways. These include Notch, Delta, receptor tyrosine kinases and homologues of the animal Toll-like receptor genes (figure 2*a*) [97,107,125,127–131]. By contrast, several upstream receptors and ligands, such as the spatial signalling genes Hedgehog, Wnt, TGF-β and JAK from the JAK-STAT network, are absent in unicellular holozoans and were likely absent from the last unicellular ancestor of animals (figure 2*a*) [95]. A similar pattern is observed among some members of the Myc–Max network [132] and the Hippo signalling pathway [108]. For example, in the latter case, some intracellular components are present in *Capsaspora owczarzaki*, whereas their metazoan upstream receptors Crumbs and Fat are animal specific [95,108]. Thus, despite several upstream receptors and ligands evolving after the transition to animal multicellularity, the last unicellular ancestor of animals already encoded several components of key metazoan signalling pathways (figure 2*a*).

A number of transcription factors (TFs) formerly thought to be animal specific are also present in unicellular holozoans. For example, several transcriptional activators of the previously mentioned Hippo signalling pathway and the Myc–Max network are present in some unicellular holozoans [100,108]. A few choanoflagellates and ichthyosporeans, as well as *Capsaspora* and *Corallochytrium*, encode LIM Homeobox TFs [24,104]. Several unicellular holozoans also encode homologues of key animal developmental TFs, such as nuclear factor-κB, the p53/63/73 family, RUNX and T-box TFs, such as Brachyury [84,95,102,109,133]. Interestingly, some of these TFs already display the potential to participate in gene regulatory networks (GRNs) well established in Metazoa, such as Brachyury and Myc [100]. This indicates that the last unicellular ancestor of animals already possessed a diverse repertoire of TFs and some of them could potentially have had similar regulatory roles to those found in animals (figure 2*a*).

Finally, a few unicellular holozoans also exhibit some of the mechanisms that animals use to regulate TF recruitment and gene expression. For example, some species encode genes involved in the control of chromatin accessibility, such as the histone acetyltransferase p300/CBP or many histone post-translational modifiers [24,100]. In *Capsaspora,* life-stage transitions are associated with changes of chromatin accessibility in only the proximal cis-regulatory regions [100]. In addition, its regulatory genome lacks animal promoter types and signatures of animal enhancers, indicating that *Capsaspora* cis-regulatory regions are small and proximal [100]. Moreover, the first evidence for post-transcriptional regulation of mRNA via miRNAs has been reported in ichthyosporeans, as some species encode several miRNA genes and homologues of the animal miRNA biogenesis machinery (including Drosha and Pasha) [134]. This indicates a unicellular origin of animal miRNAs and the associated microprocessor complex [134]. Altogether, this suggests that the last unicellular ancestor of animals likely followed a primarily proximal gene regulatory strategy and used few epigenomic mechanisms to control chromatin accessibility, which potentially could also regulate transitions between different life stages.

Thus, these findings indicate that the last unicellular ancestor of animals had a gene-rich and regulatorily complex genome. Some of the genes that were already present in the last unicellular ancestor are important for animal multicellularity-related functions, especially those involved in differential gene regulation (e.g. TFs and signalling pathways), cell adhesion (e.g. cadherins and integrins), cell-type specification, cell cycle and immunity (figure 2*a*) [34,97,122]. Nevertheless, these inferences are based on a still limited number of currently available genomes, the gene content of which varies considerably between unicellular holozoan species and lineages [41,42,97]. We expect to continue elucidating the genetic toolkit of the last unicellular ancestor of animals as more genomic data are available for more unicellular holozoans in the coming years.

#### 2.1.2. The genetic toolkit of the last common ancestor of animals

The genetic toolkit of the animal LCA can be reconstructed by comparing the genomes of extant animals. However, comparisons between extant animals and unicellular holozoans can also yield valuable insights into reconstructing the genomic features of the animal LCA [33,34,95]. Specifically, those features that are shared between unicellular holozoans and animals, which are traced back to the last unicellular ancestor of animals (see §2.1.1), can also be inferred to be present in the animal LCA (figure 2). For example, cadherins (molecules mediating cell–cell interactions), integrins (mediating cell–extracellular matrix interactions) and some basal lamina elements are shared between unicellular holozoans and most animals and are thus inferred to be present both in the last unicellular ancestor of animals and the animal LCA (figure 2) [20,22,94,135–137]. The same happens with several of the aforementioned components related to key intracellular signalling pathways and TFs (figure 2) [24–26,41,42,44,100,102]. Thus, the animal LCA also possessed key genes related to cell adhesion, signal transduction and transcriptional regulation that evolved in a unicellular context (see §2.1.1, figure 2).

Other features that are well conserved between unicellular holozoans and some animal lineages but absent in some early branching animals can also be traced back to the animal LCA [33,123]. For example, the hedgling cadherin family is inferred to have been present in the last unicellular ancestor of animals, as it is present in the genomes of some choanoflagellates, sponges and cnidarians (figure 2*a*) [33,41,42,138,139] but is absent in ctenophores, placozoans and bilaterians [33,105,138,139]. Similarly, Toll-like receptors are found in several choanoflagellate species and in nearly all bilaterians and cnidarians but are absent in placozoans and ctenophores and incomplete (i.e. partial domain architectures) in sponges [97,140,141].

Lastly, those features exclusively shared between bilaterian and non-bilaterian animals but absent from unicellular holozoans can be inferred to be present in the animal LCA. These features can be considered key animal innovations and can help identify the set of genes and mechanisms that evolved to support the fundamentals of animal multicellularity. Strikingly, most of these genes are enriched in functions of DNA binding, signalling pathways and innate immunity, as well as cell adhesion and cytoskeletal regulation [34,97,110]. For example, a key animal innovation includes the emergence of several new classes of TFs [102,110,133]. Some of these new TF classes include ETS, SMAD, nuclear receptor, Doublesex and interferon-regulatory factor TFs [110,133]. As importantly, other TF families which expanded along the animal stem (see box 1 for a definition) greatly enhanced the regulatory capabilities of the first animals. These include members of the homeobox TF family, such as Pax, Sox, basic helix–loop–helix and zinc-finger TF families [110,133]. Thus, the foundations of the animal TF toolkit were already integrated in the animal LCA (figure 2*b*).

Components of key signalling pathways also originated along the animal stem and are inferred to be present in the animal LCA. The first example includes the Wnt signalling pathway, which orchestrates cell–cell communication-mediated cooperation, specialization and polarity during animal development. For instance, frizzled, dishevelled and β- and *δ*-catenins are inferred to have been present in the animal LCA. Some of these members are indeed expressed among early branching animals, such as in sponge larvae, during cnidarian development, and in several structures of both adult sponges and adult ctenophores [136,141–145]. Others are present only in a few highly derived taxa [146,147]. Another key signalling pathway that evolved at the root of Metazoa includes the developmental TGF-β signalling pathway. Although its core components show a more scattered distribution between lineages and species across the animal tree, it is also inferred to be present in the animal LCA [20,22,141]. Similarly, many other animal signalling pathways which expanded along the animal stem (including those responsible for patterning in bilaterians and innate immunity) are present in early branching animal lineages, despite also being patchily distributed and incomplete in some species [34,141,148]. For instance, there is abundant evidence of innate immunity components occurring in different animal lineages, from Toll-like and Ig receptors to TFs and complement system in sponges and cnidarians [140,141,149–151]. Thus, the animal LCA already contained a rich repertoire of genes related to key animal signalling pathways. These key animal-specific acquisitions, especially related to members of the Wnt and TGF-β signalling pathways, are considered hallmarks of animal development and the acquisition of stable multicellularity [34,97,143,145,152].

Several genes related to cell–cell adhesion and cytoskeletal regulation also emerged at the onset of Metazoa and are inferred to be present in the animal LCA. These include, for example, Dystroglycan, Hemicentin, Fermitin [97] and the multifunctional Espin gene (figure 2*b*) [153,154]. Other components related to adherens junctions and cell polarity functions are fairly well conserved in sponges [105,136,155] with some homologues missing in ctenophores [156].

Finally, those features absent from unicellular holozoans and most non-bilaterian animals are more difficult to infer as present in the animal LCA [33,35]. An example includes the reconstruction of genes critical to the development and physiology of the nervous system [37,39,40,94]. Interestingly, some relevant genes are present in sponges, despite the apparent absence of a nervous system in this group [136,141]. By contrast, ctenophores lack neurotransmitters of the canonical nervous system toolkit present in other animals [20], leading some authors to hypothesize a parallel evolution of the nervous system in this lineage [39,40]. Nevertheless, some observations indicate that early branching animals could use this ‘simpler’ nervous system to communicate information about their microbiomes [157,158], sharing a common origin of the foundations of the neural and the immune systems at the functional level. A similar scattering pattern is observed with genes related to the development of germ layers. Ctenophores possess an independently derived mesodermal tissue, despite their lack of key bilaterian mesoderm specification genes [20,22,159]. This suggests that the regulatory mechanisms necessary for establishing early fates in layers of cells (such as the muscle cells in the ctenophore-specific mesoderm) were present before the emergence of bilaterians. If we consider ctenophores as the earliest branching animal lineage, then these mechanisms would likely have been present in the animal LCA. Thus, although the origins of the nervous system and of developmental processes remain elusive, the relevant toolkit may have existed in a simpler form in the animal LCA and later evolved into more specialized and complex systems in different lineages during animal diversification.

Overall, the emergence and expansion of key TFs and members of several signalling pathways (such as Wnt and TGF-β), as well as the evolution of elements involved in innate immunity, development and cell adhesion, were critical acquisitions that originated in the animal LCA. These systems may have helped establish the foundations of axial patterning and the acquisition of stable multicellularity in animals.

#### 2.1.3. Major forces shaping the evolution of animal genomes

Which major evolutionary mechanisms shaped the evolution of animal genomes during the transition from unicellularity to multicellularity? Previously, the innovation of some genes key to animal multicellularity was considered the most important driving force for the origin of animals. And indeed, a relatively large number of novel gene families (around 2000), which take part in processes that differentiate animals from other lineages, originated in the animal stem lineage [34,42,44,97,160]. However, only around 2% of these gene families are conserved across animal phyla, indicating that most genes originating in the animal LCA were secondarily lost in extant phyla [34,97]. Some studies estimate that the rate of gene innovation in or immediately prior to the animal LCA was larger than at other points of the animal stem. This suggests a high gene birth rate at the onset of animals which progressively decreased as animals diversified into clades [34,161]. Other studies estimate approximately equal numbers of gains and losses, finding evidence for a burst of gene family expansions in the last unicellular ancestor of animals stem (box 1), and an accelerated churn (i.e. both gains and losses, rather than only gains) of gene families that later evolved along the Metazoa stem [97,162]. In fact, a similar number of gene losses and gains are detected in animals compared to their unicellular relatives, mostly affecting pathways such as amino acid biosynthesis and osmosensing [34,97]. This points to a high turnover of genes and the potential for increased genomic plasticity during the diversification of animals, implying that a remarkable amount of gene losses and gene innovation contributed to shaping the genome composition of animals [34,97,161,163–165].

As discussed in previous sections, analyses of the genomes of extant unicellular holozoans have revealed that they indeed share an unexpectedly large repertoire of multicellularity-related genes with animals; these genes are therefore inferred to have been present both in the last unicellular ancestor of animals and in the animal LCA (figure 2) [24–26,41,42,44,45,97,98,100–104]. For instance, approximately one-quarter of the genes shared between animals and their unicellular relatives were already present in the LCA of Opisthokonta or gained at the root of Holozoa (figure 1*a* and box 1). This suggests that gene co-option of these pre-existing ancestral genes to perform new or specialized functions was an important driving force for animal origins [24,25,41,42,44,45,97,102,125,166].

The changes in gene content mentioned above were facilitated in part by two major genome expansions that contributed to gene family expansion and diversification in animals [161]. Gene family expansion and diversification specifically led to changes in the regulatory capacities of animals [34,97,110,133]. For instance, several classes of TFs also expanded to give rise to new families at the onset of Metazoa (see §2.1.2) [102,110,133]. This expansion of TFs in terms of classes and families triggered the rewiring and integration of some pre-existing core regulatory networks into more complex regulatory programmes during animal evolution [100,133]. In parallel, the evolution of non-coding genes and novel epigenetic mechanisms, such as the appearance of developmental promoters and distal enhancer elements, also increased *cis-regulatory* complexity in the animal stem lineage [100]. Finally, an additional level of acquired transcriptomic regulatory complexity, including alternative splicing events by exon shuffling, exon skipping or intron retention [24,167], also contributed to novel sources of transcriptomic innovation [24,168–171].

Overall, the evolution of animal genomes from a unicellular ancestor was made possible through a combination of ancient gene families with newly evolved genes in the animal stem lineage, shaped by an unbalanced distribution of gene gain and duplications, rampant gene family losses, gene co-option, gene family expansion and subfunctionalization (especially of several key TFs). The emergence of novel GRNs (especially distal regulatory elements such as enhancers and chromatin-structural modifications) was then a key mechanism for the evolution of animal genomes from a unicellular ancestor [24,25,34,41,42,44,45,97,100,102,110,125,136,161,166,172–174].

### 2.2. Reconstruction of the biological features of the last unicellular ancestor of animals and last common ancestor of animals

#### 2.2.1. Potential lifestyles of the last unicellular ancestor of animals

Besides analyses of their genomes, comparisons of unicellular holozoans' biological traits can also provide a comprehensive reconstruction of the cellular foundations of the last unicellular ancestor of animals. In recent years, the lifestyles and cell biology of several unicellular holozoan species have been characterized at the transcriptomic and morphological level [24–26,42,45,51,70,84,96–99,175–180]. Strikingly, each unicellular holozoan lineage features unique and distinctive traits that have changed our understanding of the biological nature of the last unicellular ancestor of animals.

**Figure 3.**
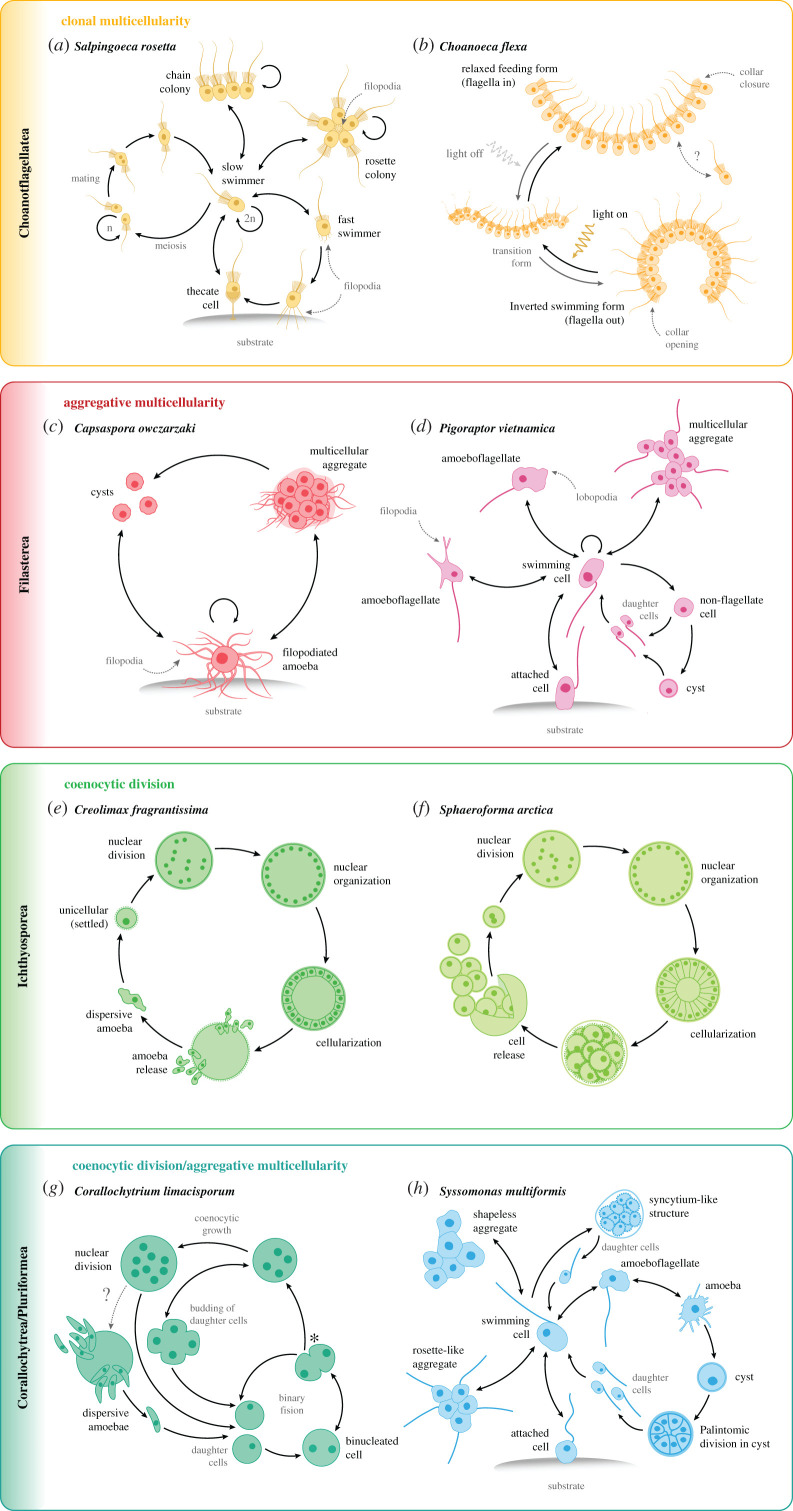
Temporally alternating life cycles of unicellular holozoans. Each panel shows life stage transitions of two unicellular holozoan species representing each clade. Arrows indicate directionality of the transition. Loop arrows indicate cell division. Dotted arrows with question marks between stages indicate potential (unconfirmed) life-stage transitions. (*a*) Life stages of the colonial choanoflagellate *Salpingoeca rosetta* [176,187]*.* The asexual life cycle (on the right) includes a single-celled sessile thecate stage (adhered to the substrate), slow and fast swimming single-celled stages, and two types of clonal colonial stages (chain and rosette colonies), in which neighbouring cells are linked by intercellular bridges [188–190]. Starvation triggers the *S. rosetta* sexual cycle (on the left), in which diploid cells (slow swimmers) undergo meiosis and recombination, and the resulting haploid cells (which can also divide asexually) mate anisogamously [176,178]. (*b*) Life stages of the colonial choanoflagellate *Choanoeca flexa* [96]*.* Light-to-dark transitions induce *C. flexa* colonies to rapidly and reversibly invert their curvature while maintaining contacts among neighbouring cells between their collar microvilli, alternating between two colony conformations. In response to light, colonies exhibit a relaxed (flagella-in) feeding form. In the absence of light, colonies transition to an inverted (flagella-out) swimming form. (*c*) Life stages of the filasterean *Capsaspora owczarzaki* [64,65,98]. In the trophic proliferative (filopodial) stage, cells are amoebae adhered to the substrate, extending several long, thin actin-based filopodia. These amoebas can detach from the substrate and actively aggregate in the aggregative or ‘multicellular’ stage, producing an extracellular matrix that presumably binds them together. In response to crowding or stress, cells from both the amoeba and the aggregative stages can encyst by retracting the filopodia into a cystic or resistance stage. (*d*) Putative life stages of the filasterean *Pigoraptor vietnamica* [26,70]. Swimming flagellated cells can form long, thin, sometimes branching filopodia that can attach to the substrate. Flagellated cells can sometimes present wide lobopodia. Flagellated cells can retract the flagellum and become roundish, to either divide into two daughter flagellated cells or transition to a cystic stage. This can, in turn, produce two flagellated daughter cells. Cells can also form easily disintegrating aggregations of cells and feed jointly. The life stages of *Pigoraptor chileana* are very similar to the ones of *P. vietnamica*, but *P. chileana* shows a much reduced capability to produce filopodia and lobopodia (both stages are extremely rare in *P. chileana*). (*e*) Life stages of the ichthyosporean *Creolimax fragrantissima* [45,77]. Single-nucleated amoebae disperse until they settle and encyst. The rounded cell undergoes multiple rounds of synchronous nuclear division (coenocytic division) without cytoplasmic division. Nuclei are later arranged at the periphery of the cell as a large central vacuole grows. Finally, the coenocyte cellularizes and new amoebas are released to start the cycle over again. (*f*) Life stages of the ichthyosporean *Sphaeroforma arctica* [99,180]*.* Single-nucleated cells undergo multiple rounds of synchronous nuclear division (coenocytic division) without cytoplasmic division. Nuclei are later arranged at the periphery of the cell. Finally, the coenocyte cellularizes, releasing a number of daughter cells to start the cycle over again. (*g*) Life stages of the corallochytrean *Corallochytrium limacisporum* [22,83,191]. Reproduction in *C. limacisporum* occurs mainly through binary fission (99% of the cases), during which a binucleated cell divides into two, symmetrical, uninucleate cells. Binucleate cells can form two lobes that can lead to cellular division (forming two monoucleate cells), or can reverse towards spherical cells. At this point (*), cells can transition to coenocytic growth (1% of the cases) and continue dividing their nuclei further forming quadrinucleated cells. Quadrinucleated cells can often form a clover-like shape (similar to bilobed cell), that generates either four mononucleate cells or returns to spherical shape and further divides to an eight, 12 and up to 32 nuclei coenocyte. Coenocytes can release dispersive amoebas to start the cycle over again. (*h*) Putative life stages of the pluriformean *Syssomonas multiformis* [26,70]*.* A swimming flagellated cell can temporarily attach to the substrate through the anterior part of the cell body or move to the bottom and transform to an amoeboflagellate form by producing both wide lobopodia and thin short filopodia. Flagellated cells can lose the flagellum via different modes and transition into an amoeba stage, which produces thin, relatively short filopodia. Both amoeboflagellate and amoeba stages can transition back to the flagellate stage. Amoeboid cells can also encyst by retracting their filopodia and rounding the cell body. Palintomic divisions may occur in the cystic stage to release several flagellated daughter cells. Flagellated cells can partially merge and form temporary shapeless cell aggregates of both flagellated or non-flagellated cells and rosette-like colonies composed by only flagellated cells (showing outwards-directed flagella). In rich medium, solitary flagellated cells can sometimes actively merge and form a syncytium-like structure, which undergoes budding and releases flagellated daughter cells.

For example, choanoflagellates are widely distributed worldwide in a range of primarily aquatic environments [89,181–186]. Despite being mostly unicellular flagellates, some species, such as *Salpingoeca rosetta,* are able to form simple multicellular structures of stably adherent cells as a result of oriented cell divisions from a single founder cell (figure 3*a*, box 1) [61,187]. Under certain conditions, *S. rosetta* flagellate cells are also able to transdifferentiate into amoeboid cells [192]. Other species, such as the recently described *Choanoeca flexa,* are able to form enormous cup-shaped colonies (figure 3*b*) [96]. Notably, these colonies reversibly invert their curvature in response to light through a rhodopsin-cGMP pathway, representing a similar behaviour to concerted movement and morphogenesis in animals [96].

Filastereans are found in freshwater, marine and animal-associated environments [25,26,50,55,64–68,70,71]. Like choanoflagellates, some filasterean species are able to form simple multicellular structures. But, in contrast to the clonal colonies found in choanoflagellates, these are formed through the active aggregation of independent cells (figure 3*c,d*, box 1) [26,67,98]. The best-described species, *Capsaspora owczarzaki,* has three different life stages, including an aggregative stage; these stages are differentially regulated at the transcriptomic, proteomic and phosphoproteomic levels (figure 3*c*) [65,98,100,101]. Others, such as *Pigoraptor* spp., are morphologically very plastic and are able to transition from amoeba and amoeboflagellate stages to cysts and aggregates of cells (figure 3*d*) [26,70].

Ichthyosporeans are found in commensal, mutualistic or parasitic relationships with aquatic (both freshwater and marine) and terrestrial animals. Most of them have been directly isolated from different animal tissues, especially guts of molluscs and arthropods [73,76–79]. Some species exhibit distinct phenotypes, such as motile pseudopodia, hyphal or plasmodial structures [76]. Ichthyosporeans also present a broadly conserved developmental mode consisting of large, multinucleated spherical or ovoid coenocytes that sometimes release multiple spherical propagules or motile limax-shaped amoebas by cellularization of the internal nuclei (figure 3*e*,*f*) [76–78,99,180,193]. Intriguingly, at least one of these species appears to generate a self-organized polarized layer of cells in the course of cellularization (figure 3*f*) [180].

Members of the Corallochytrea/Pluriformea group and *T. unikontum* also exhibit complex behaviours and developmental modes, sometimes resembling those observed in ichthyosporeans and filastereans. For example, *C. limacisporum,* is a small spherical free-living osmotroph originally isolated from marine coral reefs with a still unresolved complex developmental mode (figure 3*g*) [25,83]. Usually, cells undergo binary cell division but occasionally cell division occurs by coenocytic development followed by the release of propagules or limax-shaped amoebas, similar to ichthyosporeans (figure 3*g*) [83,191]. *Syssomonas multiformis* is a freshwater-dwelling predatory flagellate that feeds on large eukaryotic prey [26,70]. Similar to the filasterean *Pigoraptor* sp., it also has a complex developmental mode that includes amoeboflagellate, amoeboid cells, motile swimming cells, spherical cysts and sometimes clusters of multiple cells (figure 3*h*) [26,70]. Finally, *T. unikontum* is a marine free-living predatory flagellate that also feeds on eukaryotic prey [84]. Besides its flagellate form, solitary cells temporarily aggregate into flagellated or non-flagellated cell clumps as observed in *S. multiformis* or the filasterean *Pigoraptors* spp. [84].

**Figure 4.**
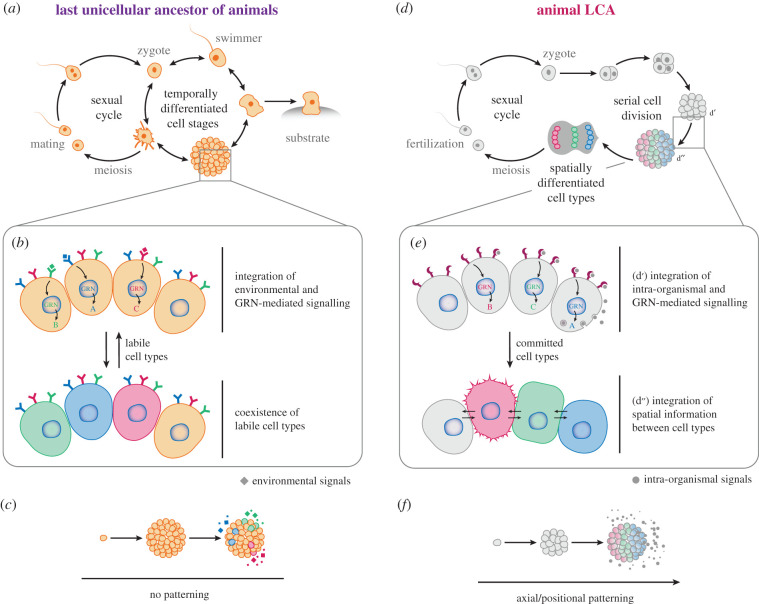
Our current perspective on important changes in the origin of animals. (*a*) The last unicellular ancestor of animals likely possessed a life cycle comprising different temporally regulated stages, including a sexually reproductive stage and at least one multicellular stage. (*b*) Cells within this multicellular structure were able to respond to different environmental stimuli thanks to a complex repertoire of signalling molecules and gene regulatory networks (GRNs), transitioning to labile cell stages. (*c*) This multicellular entity might have had a certain ability to integrate positional information from within the structure but lacked any axial/positional patterning. (*d*) The transition to animal origins likely involved some changes in this life cycle, already present by the time of the last common ancestor (LCA) of animals. (*e*) Cells within the multicellular structure acquired the ability to integrate spatial information from within the organism by making use of morphogenetic tools (such as ligands, receptors, and GRNs) (*d*′), which allowed the spatial organization of cell types (*d*″). Concomitantly, this developmental programme was conjoined with the sexual reproduction programme, by which gamete fusion was able to trigger the formation of a multicellular structure through serial division. (*f*) A greater ability to establish different cell types independently of the environment translates into the emergence of rudimentary morphogenetic plans, consisting of simple positional patterns (such as a primary axis) where different cell types localize to different regions of the organism (axial/positional patterning). It is worth emphasizing that the visual depictions presented here are mere representations of general concepts, and that we are by no means taking positions regarding specific details, such as the real structure of the life cycles, the number of cells, genes, molecules and GRNs implicated, the axial patterning or the morphological details of these organisms.

This diversity of phenotypes observed in each unicellular holozoan lineage, and the evidence of temporarily regulated life-stage transitions among some of their representatives [42,45,98,100], indicate that the last ancestral unicellular state was probably relatively plastic, rather than a simple unicellular entity (figure 4*a*) [95,123]. The last unicellular ancestor of animals could probably sense environmental stimuli and respond by transitioning to different cell stages (figure 4*a*,*b*). Its life cycle could have included a differentiated sedentary filter-feeding or heterotrophic life stage (most likely bacterivorous), and a proliferative stage, possibly including dispersive forms. It could also have included cysts or resistance forms and at least one multicellular stage. These distinct cell stages could have been regulated via temporal gene regulatory programmes, which in turn controlled life-stage transitions. Thus, the data gathered among unicellular relatives of animals suggest that the last unicellular ancestor of animals likely presented a complex life cycle integrating distinct transient cell identities, or states, and likely included a multicellular state exhibiting the spatial coexistence of different labile cell types. Future studies will provide deeper insights into whether the temporal regulation of these distinct labile cell types or stages in the last unicellular ancestor could have gradually evolved into spatio-temporal differentiation of cell types in the animal stem lineage. In fact, recent and ongoing efforts are investigating whether the multicellular structures exhibited in various unicellular holozoan species are formed by distinct cells coexisting in those multicellular stages (at the morphologic and genetic level) ([188,189,191]; S. R. Najle 2021, personal communication). If this is indeed the case, then it would suggest that spatio-temporal differentiated cell types might have been present in the last unicellular ancestor of animals.

#### 2.2.2. Potential lifestyles of the last common ancestor of animals

Comparative analyses between unicellular holozoans and animals also allow us to reconstruct the biological and ecological features of the animal LCA. In this case, those features inferred to be present in the animal LCA include traits predicted to have evolved along the animal stem. For example, the animal LCA was likely aquatic and featured obligate, clonal multicellularity [122,123]. Importantly, the animal LCA likely presented cell–cell communication-mediated cooperation, specialization and polarity, allowing the spatial distribution of labour between distinct coexisting cells. Each cell type (box 1) was specialized to perform a different role within the whole organism, with molecular features resembling those seen in the main cell types of extant animals [122]. For instance, each cell type would also have their own sets of expressed genes used in different processes (e.g. contraction, secretion, signalling and reception), regulated by well-defined genetic programmes (a set of TFs and other specific regulatory mechanisms). This implies that some genes would be expressed by certain cell types but not others (i.e. each cell type expresses a limited number of genes encoded in the genome). The genome partitioning into functional modules accessed by different cell types reflects an increase in regulatory mechanisms to determine diverse cell fates [38].

From our previous ancestral gene content reconstruction, we can also predict that the animal LCA featured cell–cell adhesion using cadherins, cell–ECM adhesion through integrin-related proteins, and orchestrated collective movement by cell contractility [123]. It also had the capacity to sense the environment, communicate between cells via synapse-like pathways and employ an epithelium-like cell layer used in part to capture bacterial or eukaryotic prey as a food source [122,123]. Moreover, it probably reproduced sexually using sperm and eggs, thus differentiating distinct gametes through spermatogenesis and oogenesis (i.e. oogamy) [122,123]. Finally, the animal LCA likely presented a form of developmental processes through mechanisms of cell division, cell differentiation and invagination present in all animals [122,123]. Such diversity of cell types and complex organization was in turn regulated by a diverse set of TFs and epigenomic machinery involving distal regulation, and the initial steps of development likely involved coordinated signalling through members of the Wnt and TGF-β pathways, paving the path to spatial distribution of labour among coexisting cells. Thus, we can conclude that the animal LCA was already rich in cell types which share some of their cellular foundations with those found in extant species.

## 3. Our current perspective on the origin of animals

The updated reconstruction of the genomic and biological features of both the last unicellular ancestor of animals and the animal LCA have allowed us to identify key features and major forces shaping animal evolution. In past, this identification was restricted by the limited information on the evolutionary relationships of animals and other eukaryotes. For instance, classical studies compared animals with unicellular organisms like yeast and designated features absent from yeast as potentially key to the origin of animals [194,195]. Now we know that such an approach was far from ideal due to the long evolutionary distances separating these lineages. In recent years, we have seen this perspective gradually changing with the study of animals’ closest unicellular relatives and their comparison to early branching animals, as discussed in previous sections. In addition, numerous studies have increased our knowledge of the environment in which animals originated and diversified. These studies have allowed us to rethink the context and major forces that drove the transition to animal multicellularity.

### 3.1. The ecological context of the transition

External factors and ecological triggers were possibly as important as genomic changes during animal evolution [34]. One example is the biogeochemical context in which animals originated and diversified. Some of the potential ecological triggers include changes in ocean chemistry, such as the availability of iron and copper [196–201] or the great oxygenation event that occurred around 700 Ma [202] (although some authors argue the latter was not as critical: [203,204]). As multicellular organisms, the origin of animals could also have been influenced by all the advantages derived from being multicellular. For example, the emergence of new ecological niches [205] and selection for multicellularity as an escape from predation were also potential driving forces for the origin of animals [206,207] (but see also [208]).

The ecological context might have also had an impact on animal evolution, such as in shaping animal feeding modes and morphological features [209]. For instance, animals evolved in an environment teeming with bacteria and other eukaryotes, and have lived in close association with these organisms throughout their subsequent evolutionary history. Indeed, host-associated microbiota can actually regulate development and gut morphogenesis in animals [157]. In this context, being in a close relationship with bacteria could have impacted animal evolution by requiring a system of cell communication to harbour bacterial symbionts and commensals, and a defence system to deal with bacterial pathogens. Interestingly, bacterial interactions are also observed among the closest unicellular relatives of animals, especially among choanoflagellates. For instance, rosette development in the choanoflagellate *S. rosetta* is known to be triggered and enhanced by a bacterial sulfonolipid [42,61,177,187,210]. Bacterial lipids also regulate developmental switches both activating and inhibiting rosette formation in *S. rosetta* [177]. This is not the sole example of environmental bacteria playing a key role during its life stages transitions, as *S. rosetta* is also capable of sexually reproducing upon induction by a bacterial chondroitinase [176–178]. Interestingly, the *S. rosetta* sexual cycle is induced by a bacterial species that also regulates light-organ development in a squid [211]. Numerous studies in other choanoflagellates highlight the role of bacterial interactions [179,212]. An example is *Salpingoeca monosierra,* a new choanoflagellate species harbouring the first known choanoflagellate microbiome [213]. *Salpingoeca monosierra* forms large colonies of more than 100 µm in diameter (more than an order of magnitude larger than those formed by *S. rosetta*) and harbour around 10 bacterial symbionts within a single colony [213]. Overall, the ecological context during animal evolution was also key for the transition to multicellularity. Living in an environment teeming with bacteria likely provided the foundations of animal-associated microbiomes and the origin of animal interactions with microorganisms.

### 3.2. The origin of animals

Besides the ecological context, former biological definitions of animals involved the capacity for cell coordination at the multicellular level, the presence of spatial cell differentiation and a coordinated developmental plan starting from a single cell. Thus, theories explaining the origins of animals involve the acquisition of mechanisms necessary to generate epithelium-like multicellular structures. Further studies and comparisons revealed that the mechanisms underpinning these features likely developed in the stem lineage of animals, building upon pathways and features present in their unicellular ancestors [24,25,45,95,98,100,122,123]. Thus, some revised theories proposed the acquisition of spatial regulation as one of the main drivers of the origin of animals, in contrast to the temporal regulation of cell types exhibited by their unicellular relatives [214,215].

We here propose an updated review of which changes might have been key to the emergence of animals (figure 4). To start with, in our view, multicellular structures with different labile cell types coexisting were likely present prior to the origin of animals. We envision an initial scenario of an ancestral organism with a complex ontogeny and temporal regulation of different transient life stages, as proposed in Zakhvatkin [215] and revised in Mikhailov [214] (figure 4*a–c*). Each stage consisted of different cell types using distinct pathways to perform specific roles, such as substrate attachment, feeding, swimming and mating. One of those stages was a multicellular structure likely originating through clonal division, displaying spatial coexistence of different, non-committed cell identities driven by unique genetic programmes of transdifferentiation (figure 4*b*,*c*). In this temporal multicellular stage, different functions (feeding, motion and secretion) occurred simultaneously as they were performed by different cells. Thus, we propose that spatial regulation itself was present in the last unicellular ancestor of Metazoa.

Below, we speculate about some aspects that may have played a key role in the origin of animals, in relation to some of their features and in no particular order, and always in the context of incremental complexity discussed in this review.

#### 3.2.1. Increased genomic innovation and co-option of pre-existing elements

The origin of animals was accompanied by increased genomic innovation, including many new, rapidly evolving and subsequently widely conserved genes. These genes encoded proteins known to have regulatory functions in animal multicellularity: gene regulation, signalling, cell adhesion and cell-cycle regulation. Nevertheless, co-option of and regulatory changes in pre-existing elements present among unicellular holozoans set the foundations for further gene family expansions and diversifications. This in turn contributed to an increased layer of regulation for cell-type specification in the animal stem lineage, and likely played a major role in the events discussed below.

#### 3.2.2. Progressive acquisition of axial patterning and cell-type identity

As previously proposed, the last unicellular ancestor of animals had a mixture of labile cell types coexisting in the same entity (figure 4*b*,*c*) [95]. However, analyses have so far not yet shown conclusive evidence that unicellular relatives of animals have specific arrangements of differentiated cell types when forming a multicellular structure. The last unicellular ancestor of animals was likely able to respond to external cues in a changing environment thanks to the signalling and genome regulatory mechanisms discussed above (figure 4*b*,*c*). Co-option of such genes for spatial cell signalling between neighbouring cells might have led to the ability to integrate positional information from within the organism. The pathways in question would involve the triggering of adjustable, non-binary responses, as in animal morphogens, and at least one mechanism of genome regulation determining different phenotypes. One potential candidate could be the Wnt/β-catenin signalling pathway, known to regulate the anteroposterior axis of the body plan even in early branching animals [142,144]. A primary axis likely arose as a result of spatial separation between different groups of cells. These primary axes could have provided a nucleating architecture for the different cell types to arrange and may have led to the formation of simple morphogenetic plans [95]. With this, spatial coordination of cells came to be equally important to define different functions in the organism, rather than just individual coexisting cells.

The integration of temporally regulated and spatially coexisting cell types could have contributed to a gradual regionalization of functions that in turn fostered the emergence of morphogenetic programmes (figure 4*d*–*f*) [95]. Flexible cell identity (and in turn GRNs) became less dependent on external factors, leading to a certain commitment of cell fate (figure 4*e*). This might have occurred through GRNs becoming more linked or dependent on signals within the organism, thereby overriding the freedom of the cell to respond to its environment by transdifferentiation. The emergence of cell types would allow selection to operate at the level of individual cells in terms of collective fitness, constituting a fine-tuning of within-group selection [216]. Inherently, the emergence of multicellular structures might have enhanced the differences between cells in different regions of this multicellular entity [217]. Thus, the transition to animal origins likely involved the progressive integration of GRNs and a gradual regionalization of functions, allowing the establishment of different spatially coexisting cell types.

#### 3.2.3. Emergence of a conjoined gene regulatory programme of fertilization and multicellular development

Animals produce very distinct kinds of gametes. Gamete fusion determines initial polarity and triggers the developmental programme in animal eggs [218,219], meaning that in earlier stages of animal evolution it could have served as an early trigger for asymmetric cell division, generation of a rudimentary axis and establishment of cell fates. During development and throughout the animal's life, animal cells are able to proliferate in response to signals from within the organism by controlling entry into the cell cycle. The set of *Capsaspora* cell cycle regulators shares some traits with those of animals, with some conserved TFs related to proliferation as well as the timing of expression of cell cycle checkpoint genes [100,220]. However, unicellular holozoans lack the genes required to trigger cell cycle progression in response to extracellular signalling in animals [220–222]. So far, we do not know of any unicellular holozoan where the formation of the multicellular stage is linked to the fusion of gametes. At some point along the stem lineage leading to animals, an ancestor with the ability to both generate a multicellular morphogenetic plan through axial patterning and perform sexual reproduction likely integrated these two programmes in a single developmental plan (figure 4).

#### 3.2.4. Relegation of unicellular stages in favour of a multicellular stage

The origin of animals likely involved a long, gradual evolutionary process rather than a single evolutionary leap, paving the way to animal multicellularity by coupling complex development, sperm–egg fusion and serial cell division in parallel with the integration of spatial cell differentiation [95,123]. The multicellular stage could have prevailed over the unicellular stage by favouring escape from predators, enhanced resource exploitation and relaxation of ecological constraints due to increases in the availability of some nutrients. The relegated unicellular stages could have later become simple forms for dispersion, or gametes, as the emerging properties concomitant with multicellularity, like the division of labour, could have led the multicellular stage to thrive as a proliferative stage [95].

## 4. New avenues of research into animal origins

The improved phylogenetic framework of animals and their unicellular relatives along with the sequencing of various omics-scale datasets has allowed an updated reconstruction of the genomic and biological features of both the last unicellular ancestor of animals and the animal LCA. These comparative studies have also highlighted various evolutionary mechanisms as important driving forces for the origin of animals. For instance, we now know that co-option of ancestral genes into new functions; expansion of pre-existing GRNs combined with the emergence of novel genomic regulatory strategies; and the progressive acquisition of spatio-temporal cell-type identities, were probably key for animal evolution. Nevertheless, many questions are still left unanswered, and further studies are needed to fully understand how those mechanisms might have impacted the transition to animal multicellularity.

For example, many genes critical for animal multicellularity-related functions have homologues in unicellular holozoans, but we still do not understand the function of these homologues in non-metazoans. In addition, some genes underwent duplications along the animal stem lineage, and their functions prior to duplication (and sub- or neofunctionalization) are not known. The functions of these genes in extant unicellular holozoans are not necessarily identical to those in the unicellular ancestors of animals; nevertheless, understanding their function in a unicellular context is essential to fully address the role of co-option during the unicellular-to-multicellular transition. In this regard, the development of genetic tools among unicellular holozoans is crucial to fully understand the function of these genes of interest and assess to which extent the unicellular holozoan orthologues perform similar or different functions in a unicellular context [223]. In recent years, our joint efforts have successfully developed transfection in several unicellular species representing all major unicellular Holozoa clades [191,193,224–227]. This tool has already provided some insights into the cell biology of several unicellular holozoans. For instance, transfection in the choanoflagellate *S. rosetta* allowed the first *in vivo* characterization of septins, a major class of cytoskeletal proteins [225]. Interestingly, the *S. rosetta* septin orthologue localized to the basal poles of the cells, resembling the localization of septins in animal epithelia [225]. Transient transfection in the filasterean *C. owczarzaki* revealed the three-dimensional organization of filopodia and actin bundles in live cells [224]. In the ichthyosporean *Creolimax fragrantissima,* transient transfection allowed tracing of nuclear divisions in a growing cell *in vivo*, and revealed that these divisions were strictly synchronized [193]. Moreover, two gene silencing strategies using RNA interference by small interfering RNAs (siRNA) and morpholinos have also been developed in *C. fragrantissima* [193]. This tool has been used to analyse the function of *c-Src* kinase animal homologue throughout its life cycle, and revealed that an existing tyrosine-specific phosphatase was potentially co-opted for the role of *Src* regulation in the highly reduced kinome of *C. fragrantissima* [131,193]. Finally, transfection has also been recently developed for two additional unicellular holozoan species: the ichthyosporean *Abeoforma whisleri* [227] and the corallochytrean *C. limacisporum* [191,228]. Both species can be transiently transfected with fluorescently tagged reporter cassettes containing endogenous genes, using the same approach developed in *S. rosetta* [191,225,227]*.* Indeed, *C. limacisporum* transfectants can also be stably maintained using antibiotic-based selection, a strategy that has allowed the reconstruction of the life cycle of *C. limacisporum* with an unprecedented level of detail [191]. More recently, CRISPR/Cas9-mediated genome editing tool has been developed for *S. rosetta,* opening new avenues of research for gene function studies using reverse genetics [226]. Under this scenario, we expect future efforts to be invested in two main directions. First, towards taking advantage of the tools developed to investigate the function of key animal ‘multicellularity-related’ genes, such as those involved in animal cell adhesion, cell communication or transcriptional regulation, in the aforementioned unicellular holozoan species. And second, towards developing genetic tools in a broader representation of unicellular holozoan species to continue expanding the functional platform of experimentally tractable systems to address animal origins.

Another important pending question concerns genome regulation in a wider representation of unicellular holozoan species. Until now, our inferences have been based on the analysis of the regulatory genome of only one single species, the filasterean *C. owczarzaki* [100]*.* Based on this study, we inferred that the last unicellular ancestor of animals probably followed a primarily proximal gene regulatory strategy, lacking some animal promoter types and signatures of animal enhancers [95,100]. However, we still need to characterize the genomic regulatory landscape of other unicellular holozoan species to accurately infer the regulatory capability of the last unicellular ancestor and fully understand how genome regulation evolved during the origin of animals. Thus, we expect future research to be directed to comparatively investigate the epigenome (including chromatin accessibility and regulatory dynamics, and transcription factor networks) of additional species representing other unicellular holozoan clades (i.e. choanoflagellates, ichthyosporeans and corallochytreans). This will allow for a more comprehensive reconstruction of the regulatory capabilities of the last unicellular ancestor of animals; address whether metazoan-like distal regulation was or was not an animal innovation; and also provide mechanistic insights into the evolution of genome regulation during the unicellular-to-multicellular transition.

We also still do not know how animal cell types appeared nor whether spatial cell differentiation was already established in a unicellular context. Although analyses in the filasterean *C. owczarzaki* revealed that some of the mechanisms required for animal spatial cell differentiation were already present in the last unicellular ancestor of animals [100], it has been assumed that spatial cell differentiation *per se* evolved at the Metazoa stem. However, we still have not investigated whether the multicellular structures exhibited by unicellular holozoans are indeed composed of morphologically and genetically identical cells or, on the contrary, they are composed of distinct cell types. Recently, the tridimensional reconstruction of rosette colonies in the choanoflagellate *S. rosetta* has unexpectedly revealed that cells within rosette colonies exhibit spatial cell disparity, varying significantly in cell size, shape and nuclear and mitochondrial content [188,189]. In parallel, microscope observations in other unicellular holozoan species, such as in the filasterean *C. owczarzaki,* have also pointed to at least different cell morphologies within the same multicellular structure (S. R. Najle 2021, personal communication). This indicates that unicellular holozoan colonies may not be just formed from the assemblage of identical single cells, but they may subsequently differentiate into distinct cell types displaying morphological modifications and, potentially, genetic modifications. Thus, we expect future studies to be directed towards analysing cell-type diversity at the genetic and morphologic level across the multicellular structures of several unicellular holozoan species representing major unicellular Holozoan clades. The integration of newly developed single-cell techniques will indeed provide a unique opportunity into these studies as they can allow to detect novel, undiscovered cell types and signatures of cell-type specific gene expression profiles [2,229–233]. Moreover, molecular data at a single-cell resolution from several animal taxa, especially among non-bilaterian animals (i.e. sponges, comb jellies and placozoans) [229–232], will also complement these studies from a comparative perspective to address animal cell-type evolution.

Finally, we also predict future research to be directed towards isolating and characterizing under-studied unicellular holozoan species. Particularly, those species falling within or related to different known unicellular holozoan clades identified from molecular environmental data, and those related to potential novel unicellular holozoan clades [86]. First, because the discovery of new unicellular holozoan species will clarify the evolutionary relationships of the tree surrounding animals. And second, because their huge diversity of morphologies, lifestyles and genetic repertoires will help us continue refining the genome content and biological features of both the last unicellular ancestor of animals and the animal LCA.

In the coming years, the development of emerging model systems among unicellular holozoans combined with the use of modern research tools will allow us to fully address these new outstanding questions with an unprecedented level of detail. We look forward to seeing advances in this field as we are now entering an exciting era in the study of the origin of animals.

## 5. Concluding remarks

In recent years, a vast body of knowledge from molecular omics has provided not only a better phylogenetic framework of animals and their closest unicellular relatives but also a better understanding of the evolutionary history of genes key to animal multicellularity. To further expand this knowledge, we must aim to improve our understanding of the closest unicellular relatives of animals from different perspectives. For instance, more genome sequences are needed to better pinpoint the origin of some genes key to animal multicellularity. Moreover, functional studies of some proteins would allow us to understand how they could have been co-opted. Efforts at the taxonomic level should also allow the identification and isolation of more unicellular holozoan species. Likewise, studying their biology through cell biological and developmental approaches might help to uncover additional aspects of their temporal multicellular stages and their potential homology to similar structures in animals. Finally, the recent establishment of genetic tools in those taxa also promises to contribute to this end. Overall, we believe the years ahead of us will be crucial to better understand this transition and we find ourselves excited about, but most importantly eager to begin, unravelling the origins of animals.

## Data accessibility

This article has no additional data.
